# Stem Cell-Based Therapeutics to Improve Wound Healing

**DOI:** 10.1155/2015/383581

**Published:** 2015-11-15

**Authors:** Michael S. Hu, Tripp Leavitt, Samir Malhotra, Dominik Duscher, Michael S. Pollhammer, Graham G. Walmsley, Zeshaan N. Maan, Alexander T. M. Cheung, Manfred Schmidt, Georg M. Huemer, Michael T. Longaker, H. Peter Lorenz

**Affiliations:** ^1^Hagey Laboratory for Pediatric Regenerative Medicine, Department of Surgery, Division of Plastic and Reconstructive Surgery, Stanford University School of Medicine, Stanford, CA 94305, USA; ^2^Institute for Stem Cell Biology and Regenerative Medicine, Stanford University School of Medicine, Stanford, CA 94305, USA; ^3^Department of Surgery, John A. Burns School of Medicine, University of Hawaii, Honolulu, HI 96813, USA; ^4^Section of Plastic, Aesthetic and Reconstructive Surgery, Johannes Kepler University, Linz, Austria

## Abstract

Issues surrounding wound healing have garnered deep scientific interest as well as booming financial markets invested in novel wound therapies. Much progress has been made in the field, but it is unsurprising to find that recent successes reveal new challenges to be addressed. With regard to wound healing, large tissue deficits, recalcitrant wounds, and pathological scar formation remain but a few of our most pressing challenges. Stem cell-based therapies have been heralded as a promising means by which to surpass current limitations in wound management. The wide differentiation potential of stem cells allows for the possibility of restoring lost or damaged tissue, while their ability to immunomodulate the wound bed from afar suggests that their clinical applications need not be restricted to direct tissue formation. The clinical utility of stem cells has been demonstrated across dozens of clinical trials in chronic wound therapy, but there is hope that other aspects of wound care will inherit similar benefit. Scientific inquiry into stem cell-based wound therapy abounds in research labs around the world. While their clinical applications remain in their infancy, the heavy investment in their potential makes it a worthwhile subject to review for plastic surgeons, in terms of both their current and future applications.

## 1. Introduction

Wound healing is a complex process involving several physiological mechanisms coordinated in an effective response to tissue injury. This process consists of several distinct, yet overlapping phases—hemostasis and inflammation, proliferation, and maturation—that result in scar formation under normal circumstances [1, 2]. Normal wound repair exists along a spectrum of outcomes resulting from tissue injury. These range from pathologic underhealing (i.e., chronic, nonhealing wounds) to pathologic overhealing (i.e., hypertrophic scars and keloids), with physiologic healing, including scar formation, somewhere in between. Interest in wound healing research continues to grow, with much focus now directed towards stem cell therapies to overcome limitations in our current wound management practices. To date, 45 published clinical studies and an additional 33 trials with as yet unpublished results have explored the potential for stem cells in addressing pathological underhealing (unpublished data). Thus, current research suggests that we are nearing a tipping point in the proliferation of stem cell-based therapies and the use of these therapies to treat disease. As such, a basic understanding of wound healing and the recent advances in stem cell therapies are important topics for plastic surgeons. Herein, we discuss the unmet need that stem cell therapies are purported to address, as well as their current uses in wound healing.

## 2. Importance of Wound Healing

The majority of the body's tissues are capable of undergoing wound repair following a disruption of tissue integrity [2]. Wound care is a major component of surgical practice both acutely (e.g., trauma, burns, and surgery) and chronically (e.g., pressure ulcers, venous ulcers, and diabetic ulcers). Upon healing, these wounds result in scar formation. Tens of billions of dollars are devoted to wound care each year [3]. Chronic wounds are especially costly, as they often require prolonged follow-up with repeated interventions and are not uncommonly resistant to therapy; it is estimated that 1% of the population at any given time is suffering from some form of chronic wound [4].

Pathological scar formation, including hypertrophic scars and keloids, is another concern in wound management. These conditions can be particularly problematic given the possibility for permanent functional loss as well as social stigma [5]. Hypertrophic scars are usually the result of traumatic injuries or burns, but surgery is another potential cause. In a given year, the 1 million burns and 2 million patients injured in motor vehicle accidents necessitating treatment, in addition to the millions of others undergoing invasive surgery, demonstrate the pressing nature of this issue [5, 6].

## 3. Normal Wound Healing Physiology

As stated previously, wound healing is comprised of three overlapping stages: (1) inflammatory phase, (2) proliferation phase, and (3) maturation phase. It is important to understand the physiological mechanisms of wound healing to fully appreciate the abnormalities underlying various wound healing disorders in order to provide adequate treatment. Here we will briefly summarize the basic physiological mechanisms of wound healing. For more in-depth discussions of these processes beyond the scope of this paper, particularly in terms of the inflammatory response, the reader is directed to reviews by Gurtner et al. [2] and Eming et al. [1].

Tissue injury initiates the wound healing response, beginning with wound hemostasis as part of the inflammatory phase. Though blood flow is restricted at the wound bed itself, the adjacent tissue is subject to increased perfusion. Inflammatory mediators are produced in concert with the coagulation cascade, generating a local concentration gradient. This promotes fibrin matrix formation and neutrophil chemotaxis. Once the matrix is established, neutrophils enter to remove the dead tissue and attempt to control any potential infections via the innate immune response. These migrating cells further amplify the inflammatory response, themselves releasing proinflammatory cytokines, contributing to the swelling and erythema often observed in the initial stages of wound healing. This phase typically lasts for 4 days [7, 8].

In the ensuing proliferation phase, inflammatory cells release various cytokines and other signaling molecules to recruit fibroblasts and vascular endothelial cells to the site of injury. Fibroblasts produce collagen, which begins to replace the provisional fibrin matrix, increasing the mechanical strength of the wound. A portion of these fibroblasts also differentiates into myofibroblasts, which contribute to mechanical wound contraction. Migrating endothelial cells contribute revascularization of the wound bed via angiogenesis, helping to support the developing granulation tissue. Keratinocytes also migrate to the wound edge, where they undergo proliferation [7, 9]. Of note, destruction of hair follicles in larger wounds correlates with slower reepithelialization secondary to the loss of the epidermal stem cell niche, potentially necessitating skin graft placement to achieve complete closure [10].

It is during the final maturation phase that the wound undergoes reepithelialization. Scar formation allows the healed tissue to regain some, but not all, of its original tensile strength. However, tissue elasticity is dramatically reduced secondary to extensive fibrosis. As the intensity of the healing response deescalates in its final stages, the majority of the endothelial cells, macrophages, and myofibroblasts localized to the wound bed undergo apoptosis. The remaining scar will continue to undergo further remodeling over the subsequent months to years [7, 11].

## 4. Targets for Novel Cell-Based Therapies

In the United States, costs of chronic wound management alone are estimated to exceed $25 billion per annum [3]. Furthermore, these therapies are often supportive with suboptimal clinical outcomes, marking chronic wounds as important targets for novel therapies. While normal wound healing results in benign scar formation, impaired wound healing processes can result in aesthetically displeasing scar formation or even a chronic, nonhealing wound. Factors understood to perturb physiological healing include aging, sedentary lifestyle (characterized by little or no physical activity), psychological status, and smoking [12]. Chronic disease states share many of the modifiable risk factors associated with poor wound healing and are themselves impediments to the physiological healing process. For example, diabetes is tightly linked to chronic wound formation in the form of nonhealing diabetic ulcers [13]. Uncontrolled diabetes impairs neutrophil and macrophage migration to the wound bed. The resultant delay of wound healing predisposes patients to develop diabetic foot ulcers, which in turn may become infected and necessitate surgical debridement or amputation. A better understanding of chronic wound pathophysiology can help identify potential roles for stem cell-based therapies in nonhealing wounds [13]. Ultimately, the goal is to create cost-effective therapies that can significantly improve the quality of life for patients suffering from these conditions. Stem cells offer a promising means to this end with the potential to heal recalcitrant wounds and prevent costly sequelae of prolonged tissue defects [14].

At the opposite end of the wound healing spectrum exists pathological overhealing, subdivided into hypertrophic scarring and keloid formation. Hypertrophic scarring is attributed to the dysregulated proliferation of inflammatory cells and fibroblasts during the wound healing process, further contributing to a highly disorganized matrix structure that is characteristic of scars [15]. Hypertrophic scarring currently has no known cure; available treatments are inadequate at curbing scar formation or diminishing the resulting aesthetic defect. Excessive inflammation is a characteristic of both hypertrophic scar formation and chronic wound beds, the latter of which have been successfully managed through stem cell immunomodulation [16]. Stem cells may thus offer a means to address pathological scarring [17].

Keloid formation is a more extreme example of pathological scar formation. Often considered as separate from hypertrophic scars in terms of their pathophysiology, histological analysis has suggested that keloids may in fact simply be further along the pathological spectrum [18]. Keloids occur solely in humans following tissue injury, not uncommonly resulting from surgical incisions [19]. Both hypertrophic scarring and keloid formation involve abnormally high levels of scar formation. However, hypertrophic scars remain confined to within the wound margins, whereas keloids invade beyond them into the surrounding normal tissue. While hypertrophic scars characteristically regress over time, keloids can grow for years and almost never spontaneously regress, leading to more devastating cosmetic outcomes [20]. In fact, the amount of scar tissue formed does not correlate with the severity of the initial wounding, so even small wounds can have substantial aesthetic consequences. Although multiple types of treatments have been attempted to manage keloid scarring, none has yielded significant results [21, 22]. However, experimental studies have demonstrated the ability of stem cells to inhibit keloid growth, opening up new avenues for their treatment [23]. Unfortunately, these findings are not universal and more study is needed in terms of stem cell applications for keloid management [24].

## 5. Traditional Approaches to Wound Healing

In cases where tissue defects necessitate placement of a skin graft, surgeons can ideally utilize autologous tissue, foregoing any need for immunosuppression. However, autograft harvest is not possible in all cases, for example, due to insufficient tissue for harvest. In scenarios that preclude autologous tissue grafting, surgeons can utilize cadaveric tissue, termed allografts, or porcine xenografts. These are merely temporizing measures to provide growth factors for wound healing, as the host immune response causes transplant rejection in the weeks following implantation [25].

Tissue availability and graft immunogenicity are common issues in all areas of transplant medicine. Skin grafting is no exception, spurring the development of tissue engineered skin substitutes. The first of these substitutes were known as matrix-based products, which continue to be used today. These matrices are implanted at the wound bed, where they function as templates for revascularization and dermal regeneration. However, complete wound healing still often necessitates epidermal covering of the neodermis by skin graft or flap, though some small defects can be left to heal by secondary intention [26]. More recent developments in tissue engineering have led to the application of cell-based therapies. As opposed to harvesting areas of dermal tissue, keratinocytes can now be harvested from patients. Subsequent* ex vivo *expansion thereby allows for the production of an autologous epidermal graft. However, the product is very thin, fragile, and relatively expensive to produce [26].

It is clear that there have been multiple attempts to increase the effectiveness of wound healing techniques, as well as creating more efficient and reliable grafts. Unfortunately, even the most advanced engineered skin substitutes demonstrate limitations; they are very expensive, are not always effective, and cannot completely reconstitute skin appendages. A different approach to wound healing is therefore necessary to overcome current barriers in wound therapy and create more pragmatic and effective solutions to wound-related issues [27]. The pluripotent nature of stem cells suggests that they may provide a means to overcome at least some of the aforementioned barriers to optimal wound management.

## 6. Stem Cells and Wound Healing

**Figure 1:**
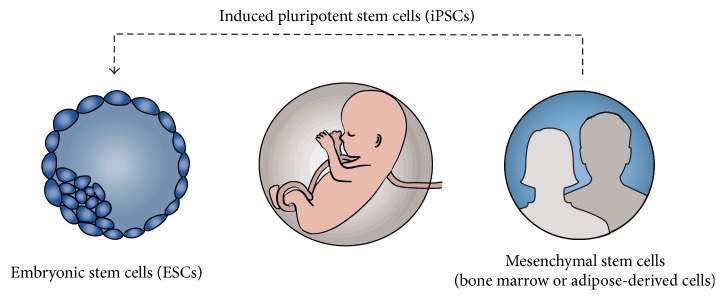
Stem cell populations.

In order for cells to be classified as stem cells, they must fulfill two criteria: they must have a prolonged capacity for self-renewal and they must be able to employ asymmetric division to differentiate into more specialized cell types [28]. These characteristics endow a set of unique abilities in these types of cells, which could be harnessed to aid the regeneration and repair process in damaged skin. Studies using models of tissue injury have shown severe injury has resulted in a dramatic increase in the number of stem cells circulating in blood [29]. Furthermore, circulating bone marrow-derived cells were found to localize to the wound site where they also differentiated into nonhematopoietic skin structures [30]. Other such findings also suggest that stem cells play a very important role in the process of wound healing, and further studies are needed to better understand the underlying mechanisms. This section will elaborate on notable findings in wound healing applications of various stem cell populations (Figure 1), such as mesenchymal stem cells (MSCs) (including adipose-derived stem cells (ASCs)), induced pluripotent stem cells (iPSCs), and embryonic stem cells (ESCs).

The majority of studies looking at potential stem cell-related wound healing therapies have centered on adult stem cells, specifically mesenchymal stem cells (MSCs). MSCs are able to self-renew and have shown great promise for treating tissue damage involving immune responses [31, 32]. MSCs can be harvested from a patient's bone marrow, adipose tissue, umbilical cord blood, and dermis [33]. Not only do autologous MSCs forgo the risks of transplant rejection, they are also understood to inhibit the inflammatory response at the wound bed, which can otherwise impair effective tissue regeneration [32, 34]. Moreover, bone marrow-derived MSCs (BM-MSCs) have been shown to synthesize higher amounts of collagen, growth factors, and angiogenic factors than the native dermal fibroblasts, which suggests that they could be implanted in wounds to increase the rate of healing without eliciting any immune response. One case study also demonstrated closure of a recalcitrant diabetic foot ulcer treated with a combination of direct BM-MSCs to the wound bed covered with a biograft composed of autologous skin fibroblasts in a collagen membrane [35]. Infection also often complicates management of chronic wounds, presenting a further issue to address in treatment. Another mechanism by which MSCs can augment the wound healing response is via antimicrobial peptide secretion [36]. In targeting numerous aspects of wound healing, stem cells thus offer a versatile treatment for wounds that have not responded to standard care.

Though MSCs have demonstrated a consistent ability to increase the rate of wound healing in a variety of scenarios, there are still some drawbacks to these therapies. For example, MSCs are a practical approach to small wounds, but it is unfeasible to culture enough MSCs to apply to a large wound. In addition, the population of MSCs within humans decreases over time, possibly eliminating the option of using autologous MSCs for treatment in the older generations [37]. While MSCs have been observed to directly contribute to wound healing via transdifferentiation into keratinocytes [38], paracrine mechanisms are generally believed to play a much more important role [39]. Therefore, fewer cells may be required for clinical efficacy, circumventing potential limitations for stem cell-based wound therapies and maintaining them as exciting modalities to improve wound healing.

While surgical manipulation and harvest of adipose tissue are generally simple procedures, the tissue itself is complex. Adipose tissue is comprised of a plethora of cells including adipocytes, smooth muscle cells, fibroblasts, macrophages, endothelial cells, and lymphocytes, as well as adipose-derived stem cells (ASCs). ASCs are a class of MSCs, pluripotent cells able to differentiate into bone, cartilage, tendon, and fat, provided they are cultured under the necessary conditions. They share an almost equal potential with MSCs to differentiate into cells of mesodermal origin but are preferred due to their wide availability and relative ease of harvesting sufficient cell numbers [40]. ASCs have been shown to promote human dermal fibroblast proliferation at the wound site by secretion of paracrine factors, which ultimately increase the rate of wound healing [41]. Another study showed that ASCs, under hypoxic conditions due to inflammation, significantly increase levels of collagen synthesis and help reduce wound area. Further study showed that this was achieved by upregulation of imperative growth factors, vascular endothelial growth factor (VEGF) and basic fibroblast growth factor (bFGF) [42]. Such evidence demonstrates the immense promise of ASCs in future wound management.

Several issues have arisen in terms of MSC and ASC use. The small population of available MSCs and necessitation of painful invasive harvest procedures have in part been circumvented by shifting to ASC applications [43]. However, a number of other issues remain. The efficacy of any cell-based therapy requires that sufficient numbers of cells be administered, which has often led to* ex vivo* expansion of MSCs for clinical use. This may be problematic as long-term culture can result in epigenetic and phenotypic changes in cell populations, potentially affecting efficacy or worse, resulting in harmful mutations [44]. Closed-system bioreactors offer a means to increase cell numbers and reduce variability of culture methods, increasing the potential for large-scale clinical use [45]. Given the challenges of* ex vivo* stem cell culture, in addition to findings that once transplanted, MSC survival is often short-lived and their effects transient, technologies to improve their efficiency are also heavily sought after [16]. Various developments have occurred to improve means of administering cells, such as within fibrin sprays [46]. Enhancing the local microenvironment of transplanted stem cells, for example, by seeding them in human collagen matrices [47, 48], provides a means for optimization of cell delivery and survival. Stem cell enhancement is not limited to collagen scaffolds, as hydrogels and silk fibroin scaffolds have also improved wound healing characteristics of coadministered stem cells [49, 50]. New methods for targeting stem cells to desired tissues with peptide or antibody marking could potentially eliminate the need for direct administration [51]. Harnessing the potential of stem cells in wound therapy has created vast opportunities for innovation, in terms of both basic science research and commercialization of new technologies. As cell therapies continue to be optimized, more applications of adult stem cells such as ASCs and MSCs will be developed for use by plastic surgeons.

The astounding proliferative capacity of the embryo suggested that the study of embryonic stem cells (ESCs) might further our understanding of regenerative processes and provide more optimal wound treatments. While embryos had originally been regarded as a key source of pluripotent stem cells, ESCs have been a topic of extreme controversy in the United States, and access to these cells in the past has been very limited. ESCs are derived from the inner cell mass of the blastocyst, an early-stage preimplantation embryo. Thus, ESCs cannot be harvested from the patient and their direct use would involve all the drawbacks of allografting, in addition to ethical concerns associated with embryonic tissue [52]. While ESCs themselves are less suitable for tissue grafting, they do provide the potential to augment physiological healing processes via paracrine mechanisms. For example, ESC-derived endothelial cells secrete a variety of cytokine factors leading to enhanced wound healing [53].

Finally, the landmark study conducted by Takahashi and Yamanaka in 2006 described a method of reprogramming adult cells back to an embryonic state, termed induced pluripotent stem cells (iPSCs) [54]. These cells opened up many new avenues in stem cell research by circumventing ethical controversy and issues associated with exogenous tissue rejection. One study managed to reprogram dermal fibroblasts into iPSCs, without use of a viral vector, which meant that iPSCs could be derived for the sick and/or elderly patients who most likely need them more [55]. Another study has shown that iPSC-derived fibroblasts show an increased production of extracellular matrix proteins that could also increase the rate of wound healing [56]. The role of iPSCs continues to expand across numerous fields of research, from the basic to translational sciences. In 2014, a Japanese team became the first to administer iPSCs clinically, in this case for the treatment of age-related macular degeneration. However, reliable iPSC-based therapies for wound management remain elusive, in part as we continue to await the results of their first clinical application. The administration of dedifferentiated pluripotent cells carries risks of subsequent tumor formation and thus long-term preliminary studies must be conducted prior to any proliferation in terms of their clinical use. We must continue to expand our understanding of how they can modulate the wound environment, while also improving our ability to manipulate them* in vitro *and* in vivo*. In this manner we can more effectively translate our discoveries from bench to bedside.

Issues pertaining to wound healing demonstrate a significant burden to the healthcare system as a whole, but their negative psychosocial impact on patients is immeasurable. Traditional wound healing technologies, including skin grafting and tissue engineered skin substitutes, remain invaluable in clinical practice. However, the growing prevalence of recalcitrant wounds goes hand-in-hand with the rise of chronic disease. It is thus imperative that older wound management techniques are augmented with novel cell-based therapies to address the limitations of current treatments.
